# Supplementary figures and images for: The CD73 immune checkpoint promotes tumor cell metabolic fitness
Source: eLife. 2023 Jun 1;12:e84508. doi: 10.7554/eLife.84508 (PMC10259490; doi:10.7554/eLife.84508)

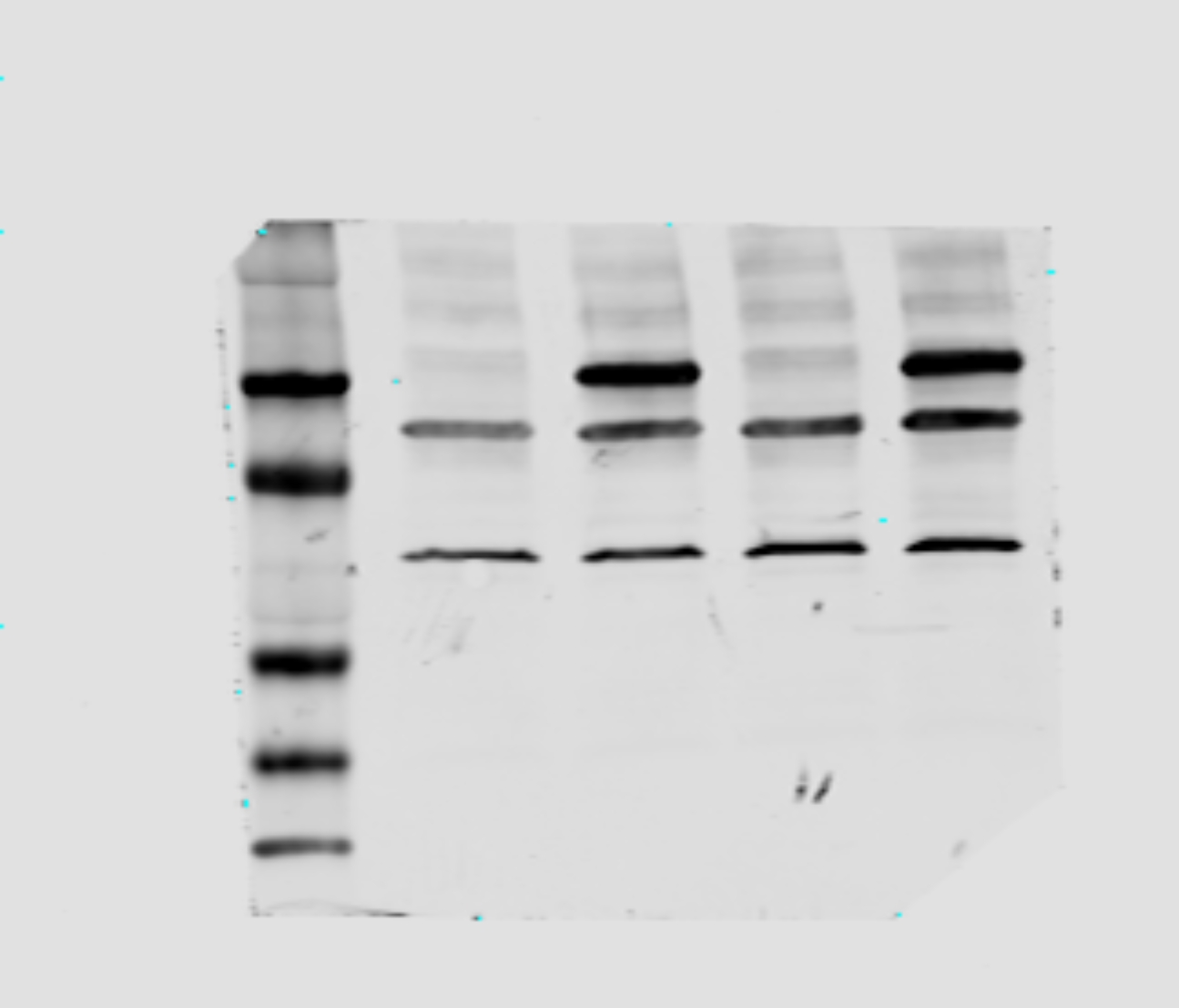

Supplement: Figure 1—source data 1. [file elife-84508-fig1-data1.zip › Fig1G_FLAG.jpg]

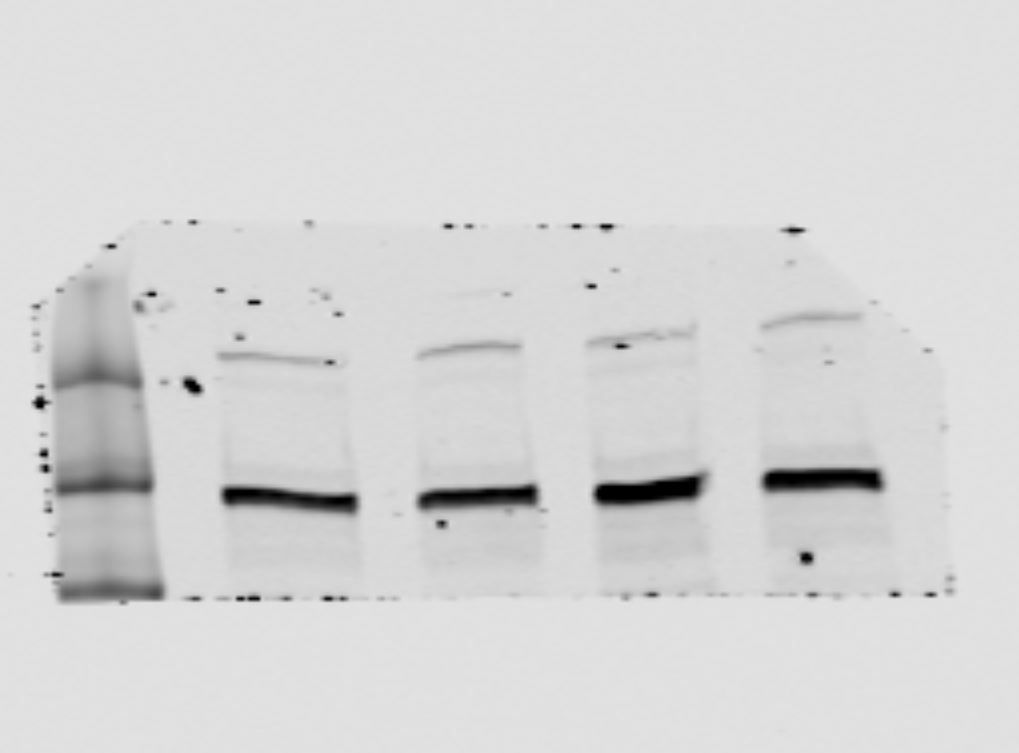

Supplement: Figure 1—source data 1. [file elife-84508-fig1-data1.zip › Fig1G_PLCg.jpg]

Fig1G

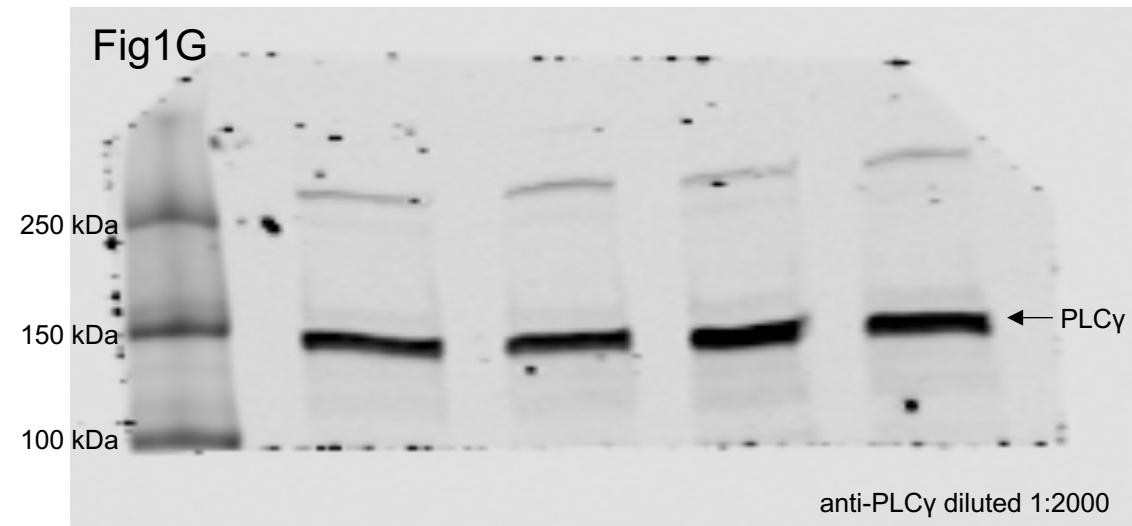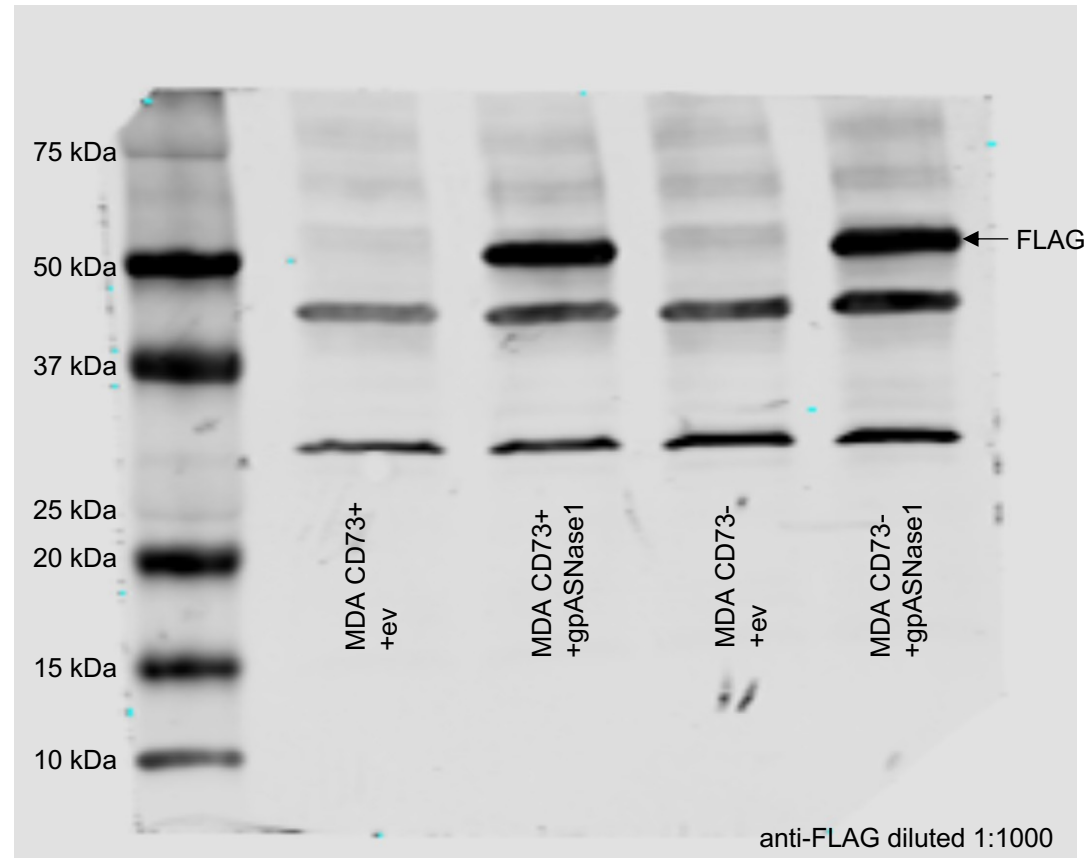

Supplement: Figure 1—source data 1. [file elife-84508-fig1-data1.zip › Fig1G_source-data.pdf]

Fig1-supp1B

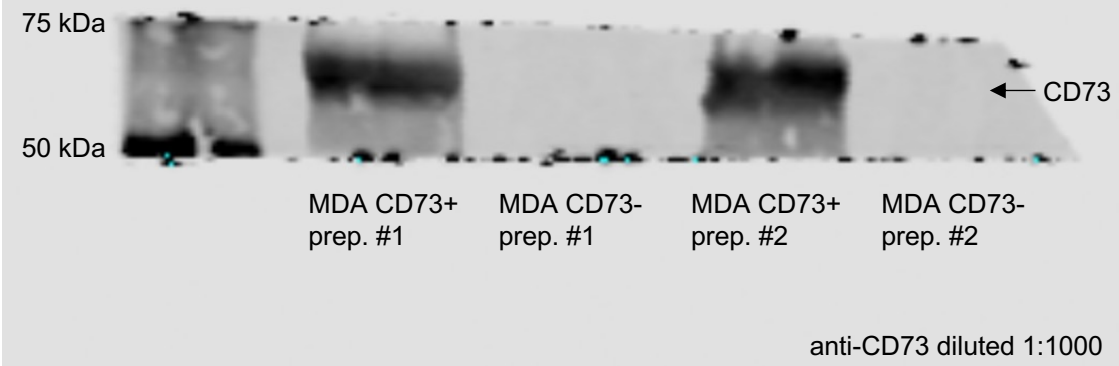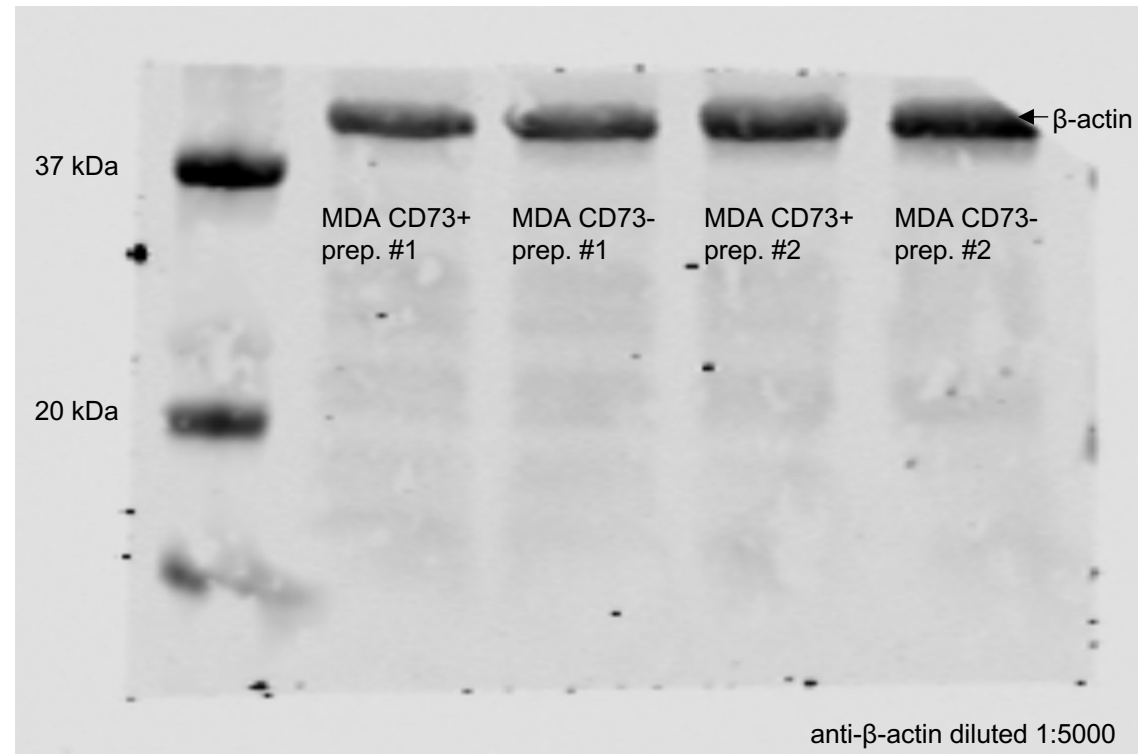

Fig1-supp1D

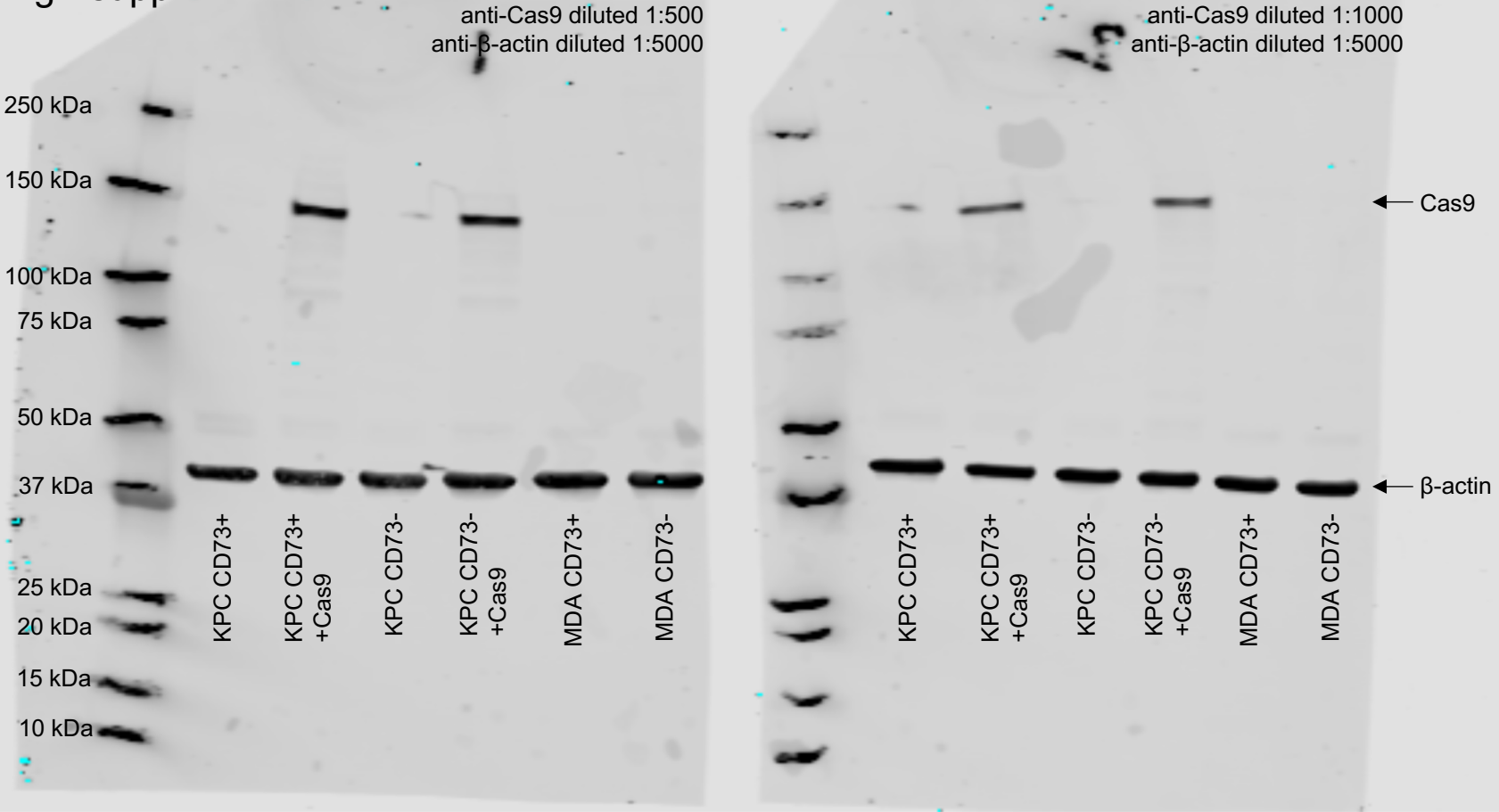

Supplement: Figure 1—figure supplement 1—source data 1. [file elife-84508-fig1-figsupp1-data1.zip › Fig1-S1_source-data.pdf]

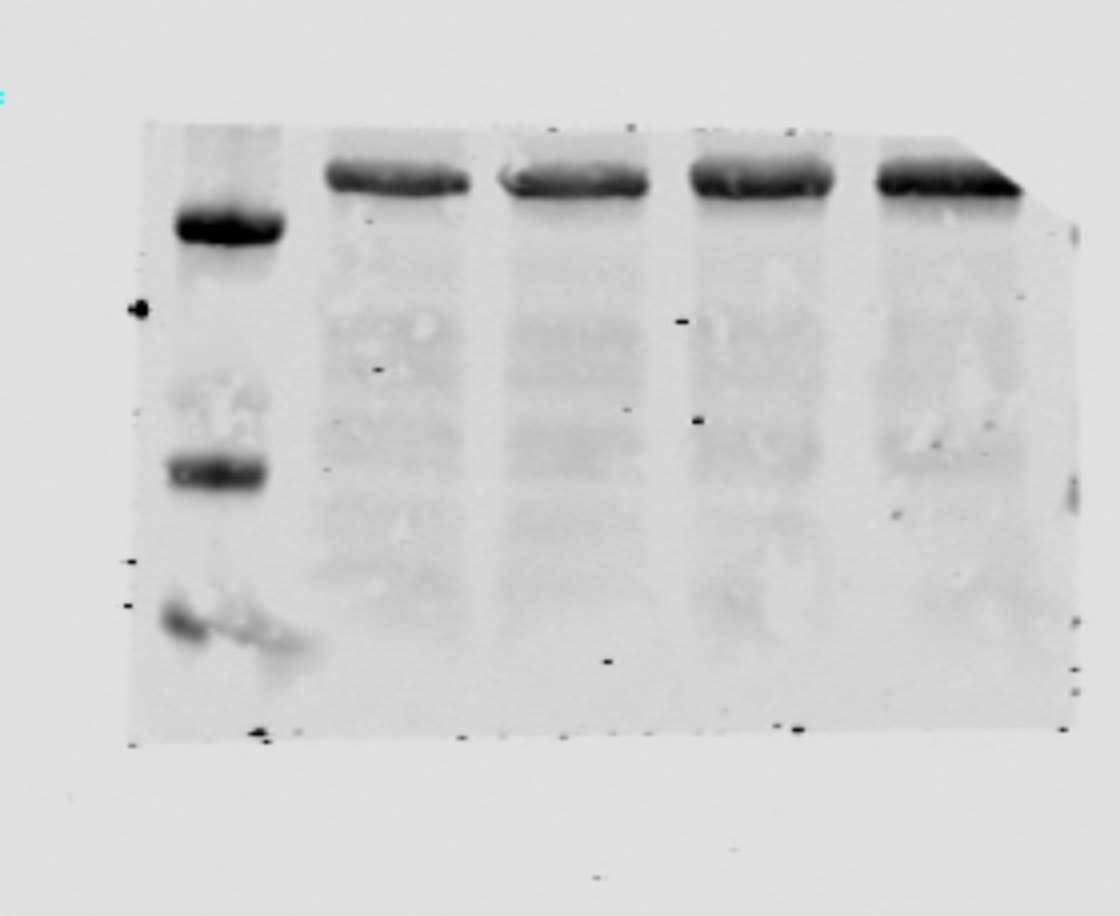

Supplement: Figure 1—figure supplement 1—source data 1. [file elife-84508-fig1-figsupp1-data1.zip › Fig1-S1B_ActB.jpg]

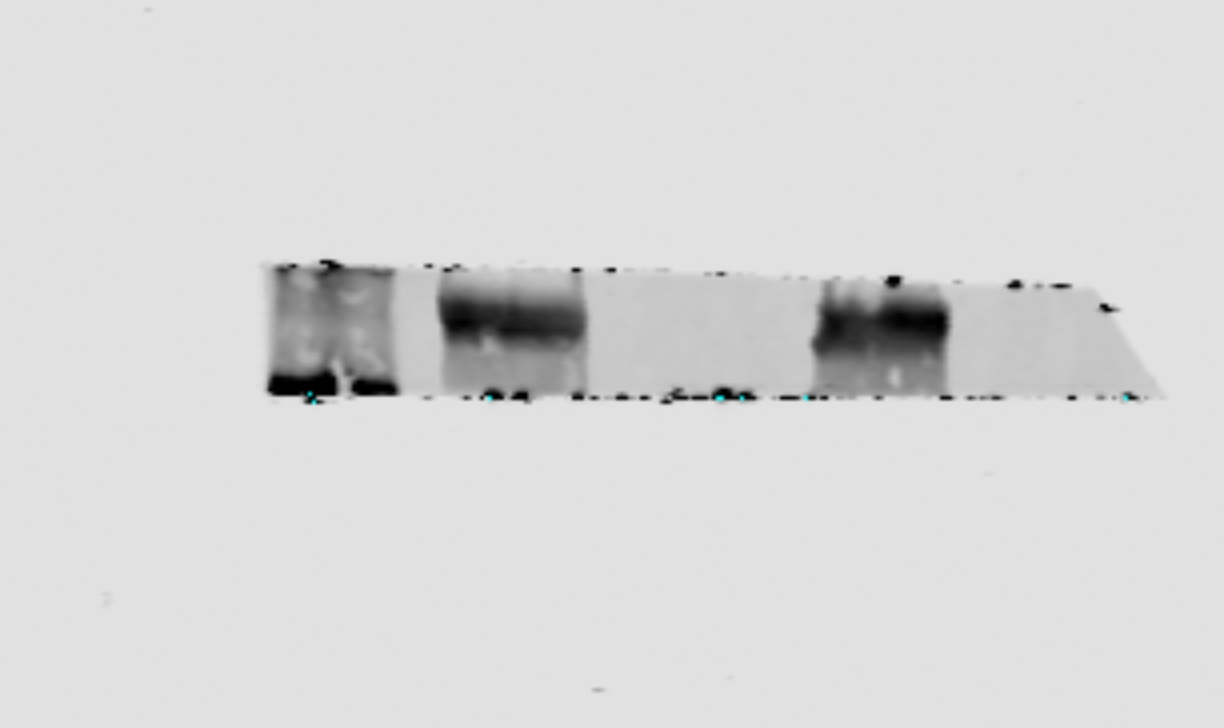

Supplement: Figure 1—figure supplement 1—source data 1. [file elife-84508-fig1-figsupp1-data1.zip › Fig1-S1B_CD73.jpg]

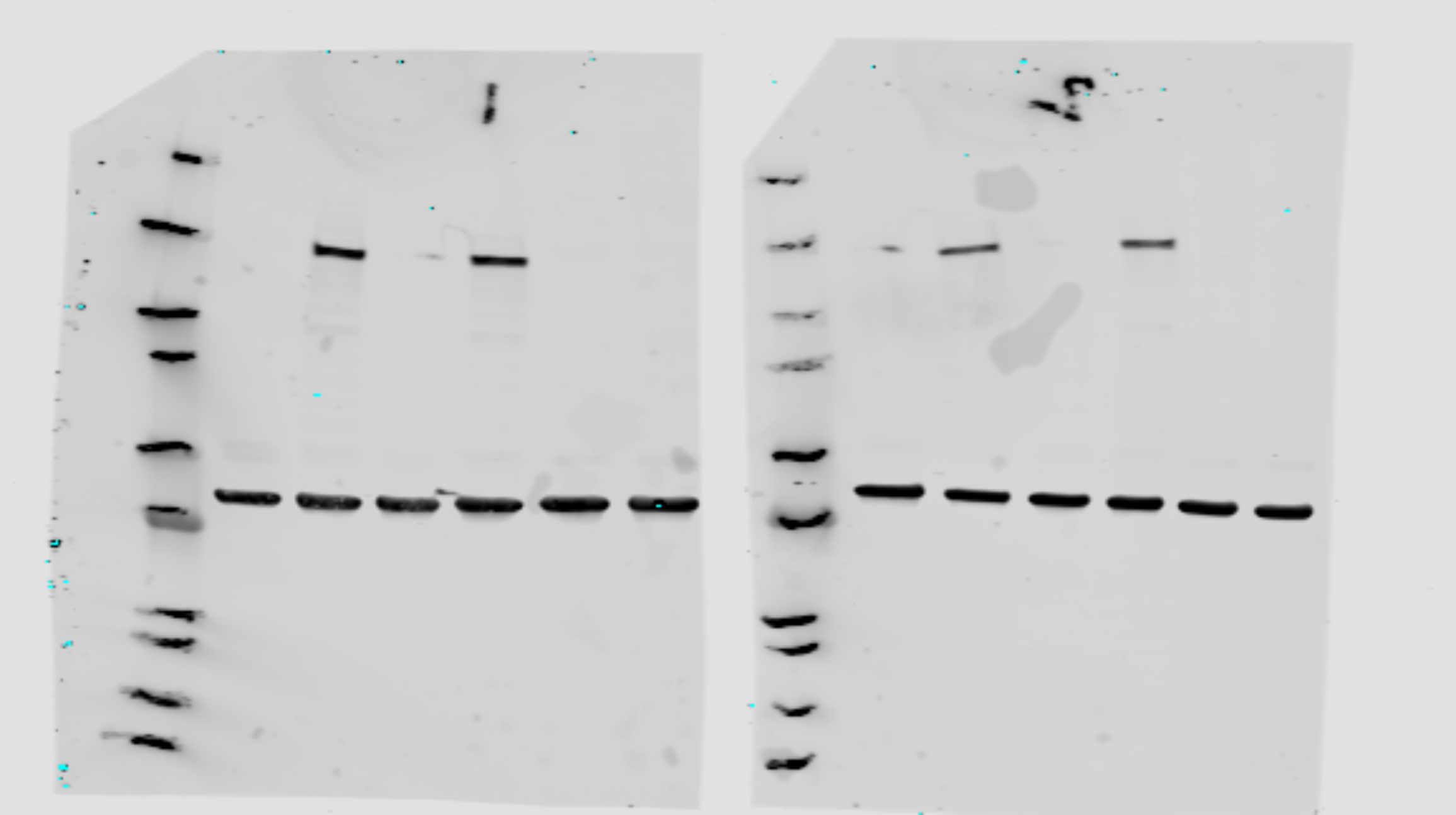

Supplement: Figure 1—figure supplement 1—source data 1. [file elife-84508-fig1-figsupp1-data1.zip › Fig1-S1D_Cas9-ActB.tif]

Fig4A

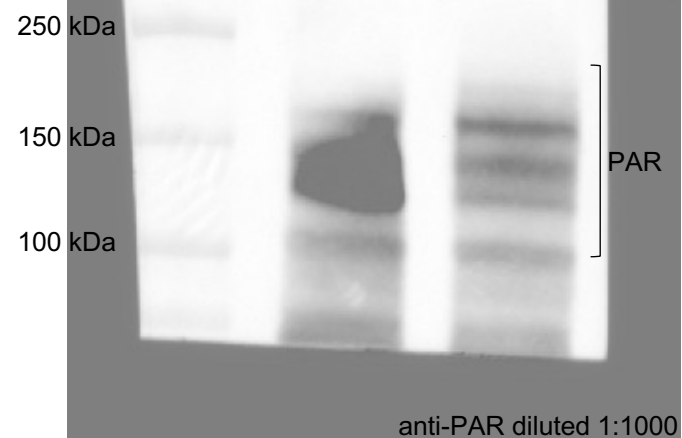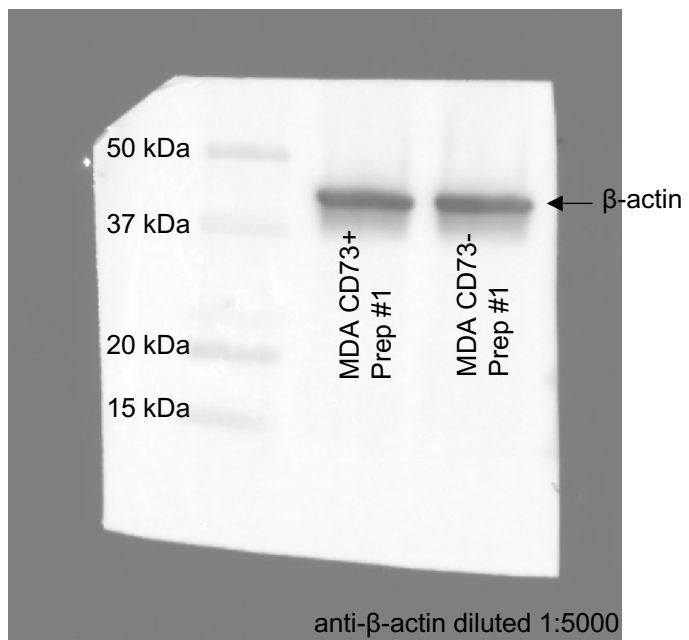

Fig4B

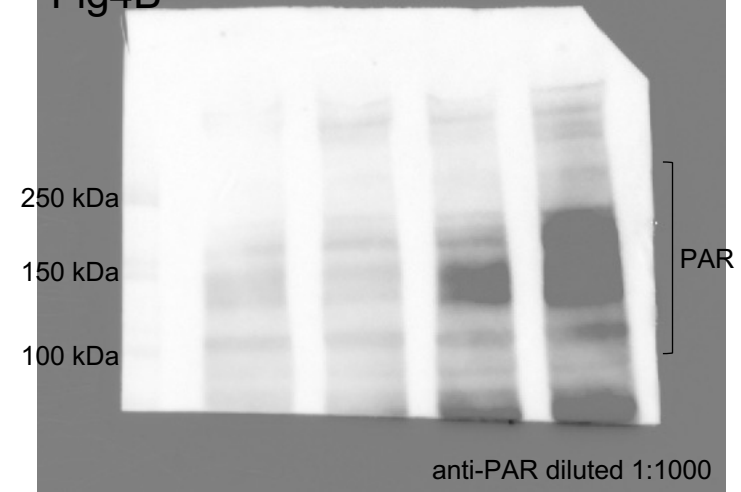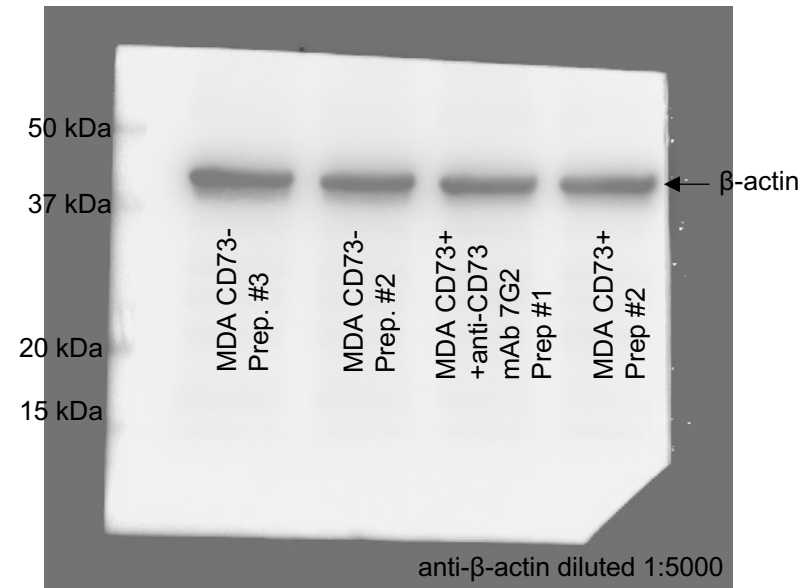

Fig4C

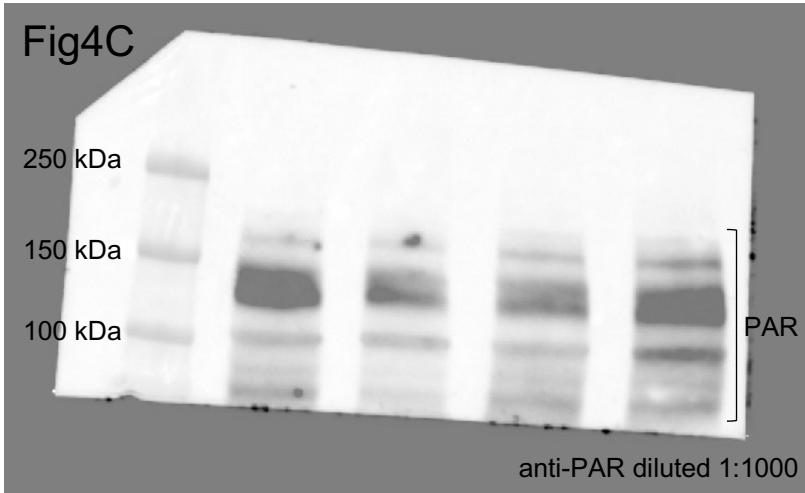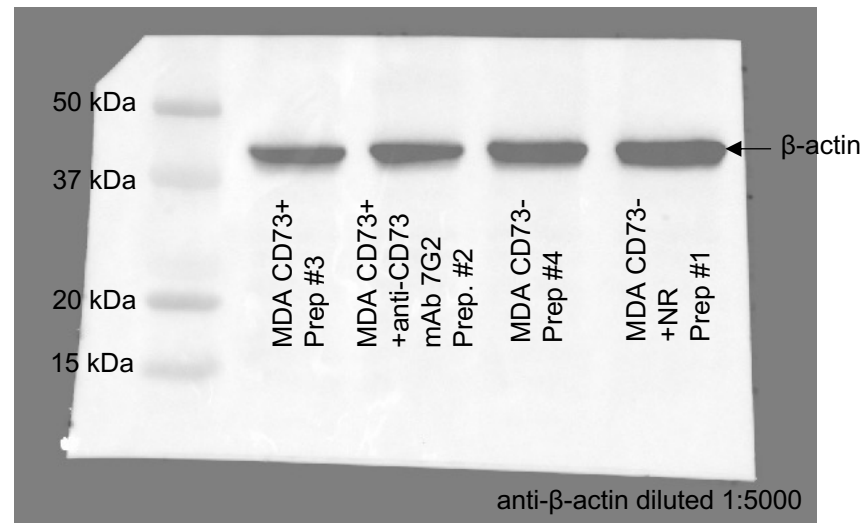

Supplement: Figure 4—source data 1. [file elife-84508-fig4-data1.zip › Fig4_source-data.pdf]

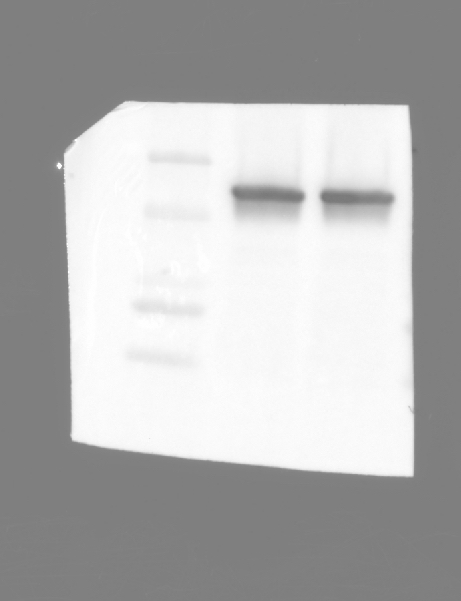

Supplement: Figure 4—source data 1. [file elife-84508-fig4-data1.zip › Fig4A_ActB.jpg]

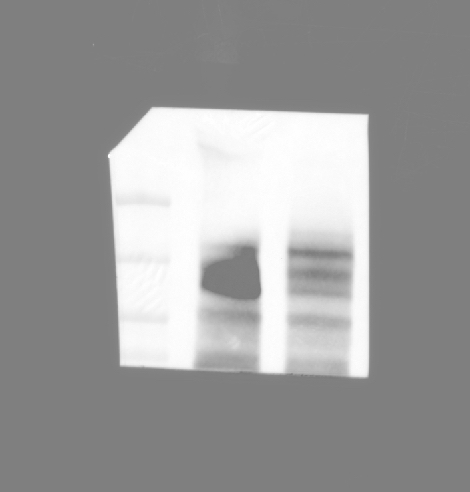

Supplement: Figure 4—source data 1. [file elife-84508-fig4-data1.zip › Fig4A_PAR.jpg]

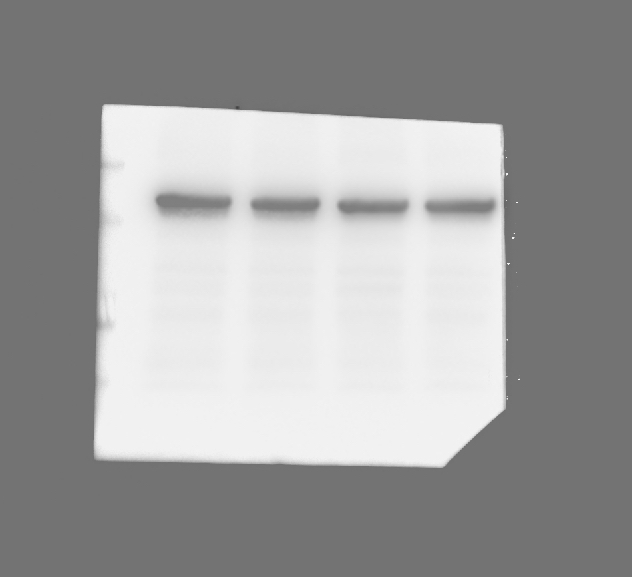

Supplement: Figure 4—source data 1. [file elife-84508-fig4-data1.zip › Fig4B_ActB.jpg]

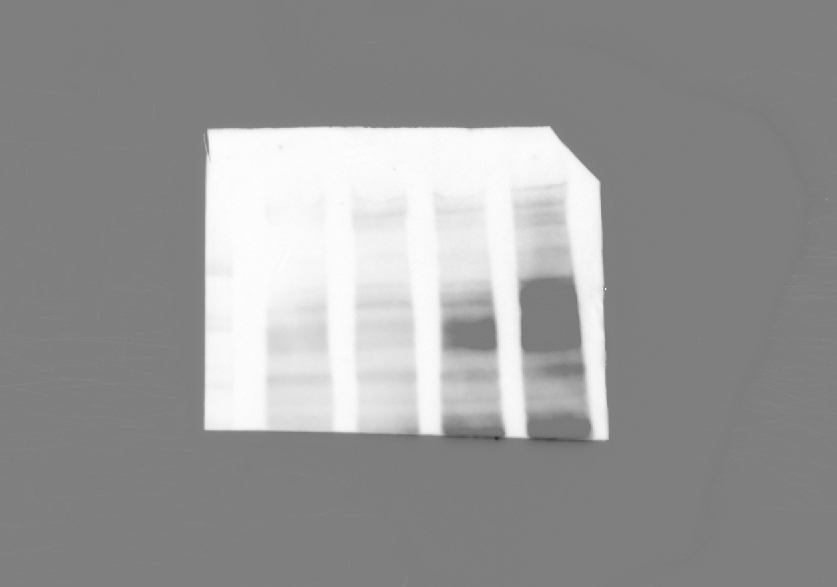

Supplement: Figure 4—source data 1. [file elife-84508-fig4-data1.zip › Fig4B_PAR.jpg]

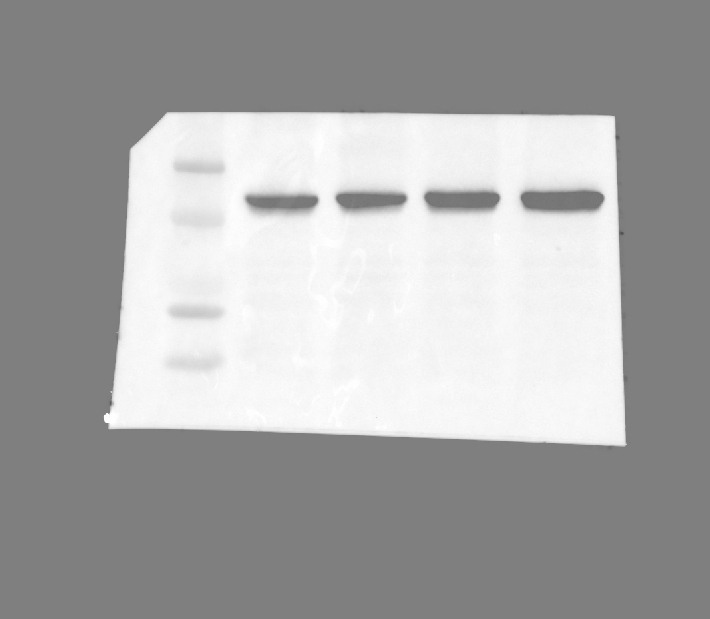

Supplement: Figure 4—source data 1. [file elife-84508-fig4-data1.zip › Fig4C_ActB.jpg]

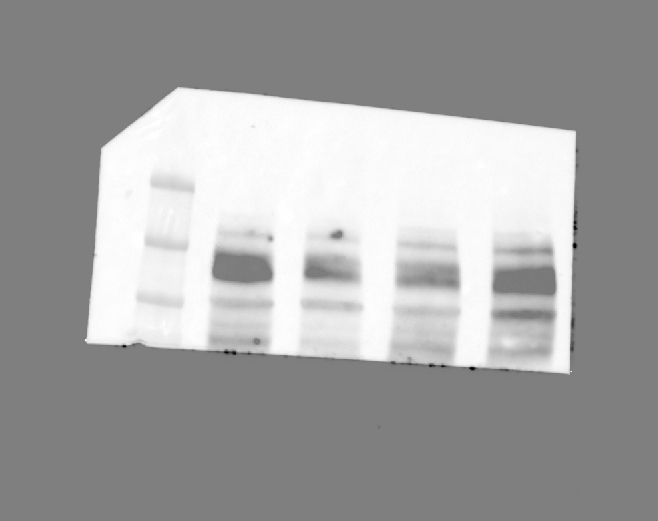

Supplement: Figure 4—source data 1. [file elife-84508-fig4-data1.zip › Fig4C_PAR.jpg]

Fig4-supp1B

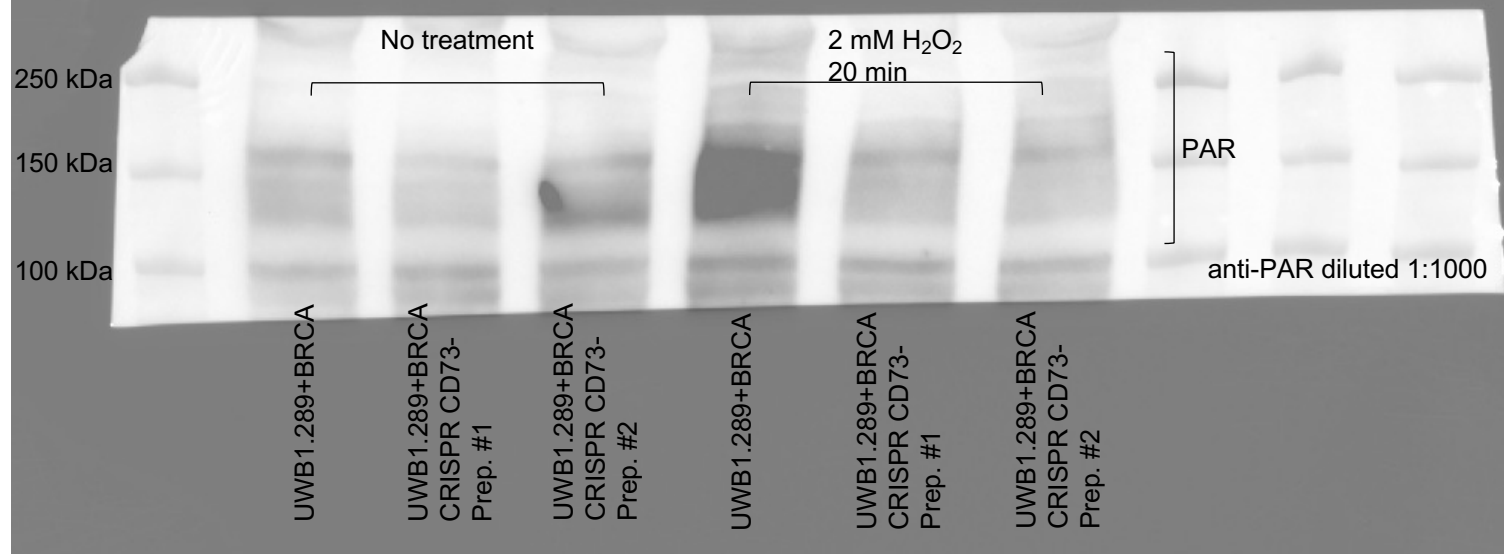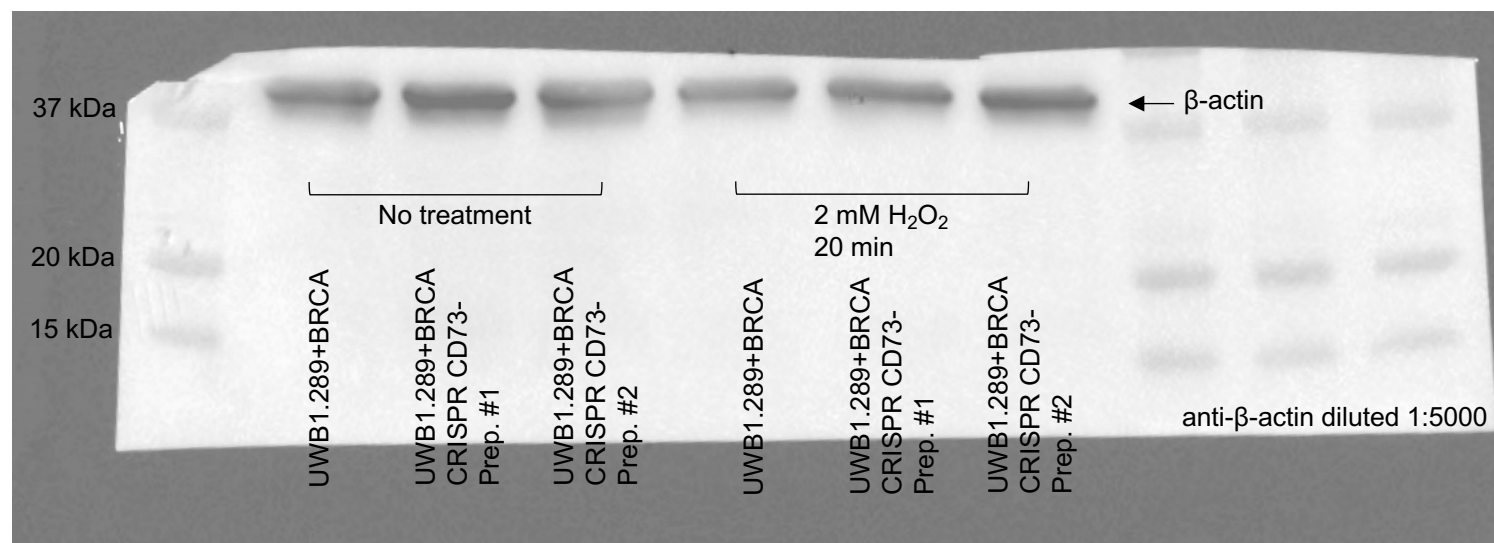

Supplement: Figure 4—figure supplement 1—source data 1. [file elife-84508-fig4-figsupp1-data1.zip › Fig4-S1_source-data.pdf]

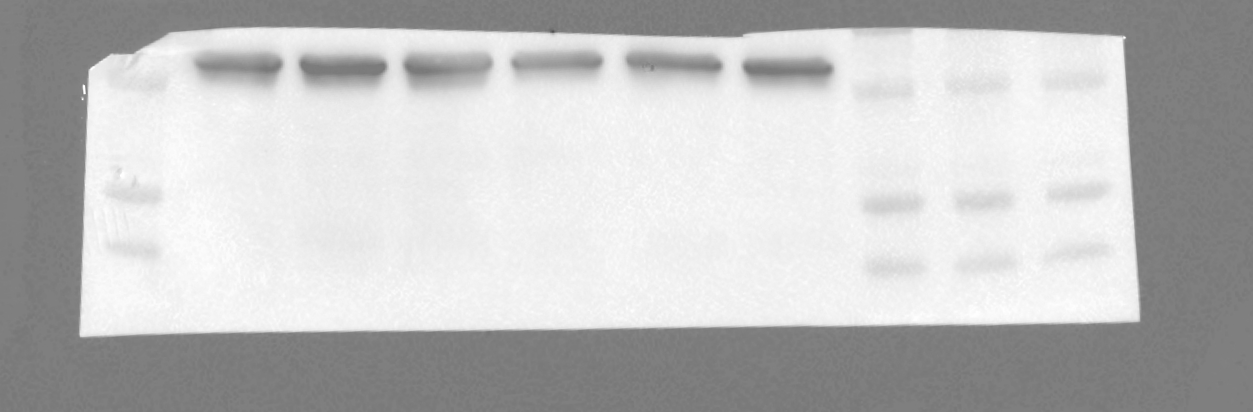

Supplement: Figure 4—figure supplement 1—source data 1. [file elife-84508-fig4-figsupp1-data1.zip › Fig4-S1B_ActB.png]

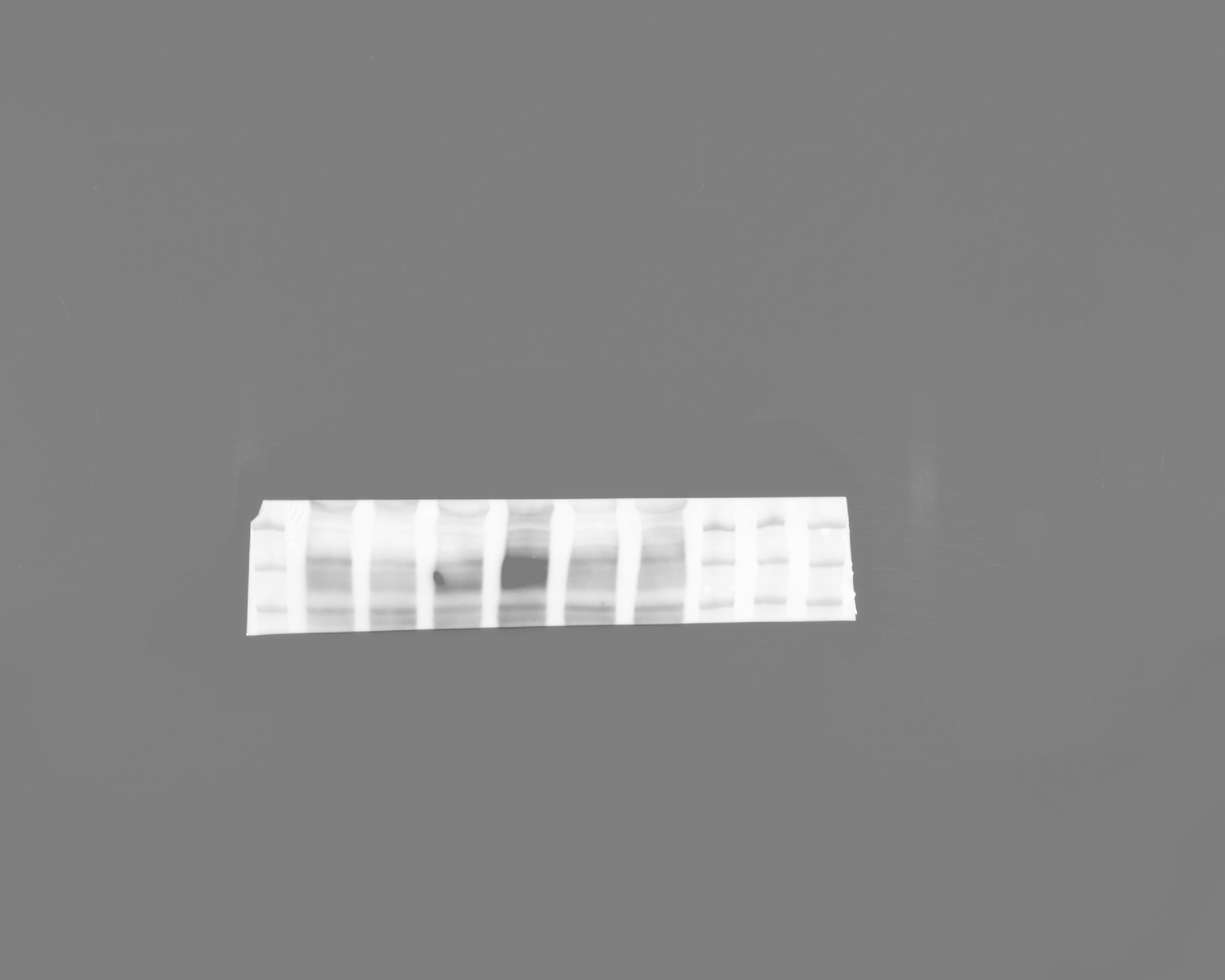

Supplement: Figure 4—figure supplement 1—source data 1. [file elife-84508-fig4-figsupp1-data1.zip › Fig4-S1B_PAR.jpg]
